# Concentration of SARS-CoV-2-Infected Cell Culture Supernatants for Detection of Virus-like Particles by Scanning Electron Microscopy

**DOI:** 10.3390/v14112388

**Published:** 2022-10-28

**Authors:** Marion Le Bideau, Lea Robresco, Jean-Pierre Baudoin, Bernard La Scola

**Affiliations:** 1 Institut de Recherche pour le Développement (IRD), Aix-Marseille University, Microbes Evolution Phylogeny and Infections (MEPHI), 27 Boulevard Jean Moulin, 13005 Marseille, France; 2 Assistance Publique-Hôpitaux de Marseille (AP-HM), 264 Rue Saint-Pierre, 13005 Marseille, France; 3 IHU Méditerranée Infection, 19-21 Boulevard Jean Moulin, 13005 Marseille, France

**Keywords:** virus, cell culture, diagnostic, scanning electron microscopy, SARS-CoV-2

## Abstract

There is currently a need for new rapid viral diagnostic electron microscopy methods. Although the gold standard remains the transmission electron microscopy (TEM) negative staining method for electron microscopic examination of samples containing a virus, difficulties can arise when the virus particle content of the sample that has to be examined is poor. Such samples include supernatants of virus-infected cells that can be difficult to examine, as sometimes only a few virus particles are released in the culture medium upon infection. In addition to TEM, scanning electron microscopy (SEM) can also be used for visualizing virus particles. One advantage of SEM over TEM is its ability to rapidly screen several large specimens, such as microscopy slides. In this study, we investigated this possibility and tested different coating molecules as well as the effect of centrifugation for analyzing SARS-CoV-2-virus-infected cell culture supernatants deposited on microscopy glass slides by SEM. We found that centrifugation of 25XConcanavalinA-coated microscopy glass slides in shell vials provided an improved method for concentrating SARS-CoV-2-virus-infected cell supernatants for virus-like particle detection by SEM.

## 1. Introduction

There is currently a need for new rapid virus screening methods using microscopy [1], especially for diagnostic electron microscopy (DEM) [2,3]. For the microscopic examination of samples containing a virus, either directly from patients or from cell cultures, the gold standard method remains transmission electron microscopy (TEM) negative staining. This consists of depositing a drop of virus-rich sample on a membrane located on a grid, counterstaining it with heavy metals and examining it using TEM [4,5,6,7,8,9,10,11,12,13,14,15]. Methods such as this are very powerful and TEM has proven its strengths over the years since Helmut Ruska’s pioneering work in the electron microscopy of viruses [16,17], revealing the morphology of virus particles and helping with their identification. However, difficulties can be faced when the sample that has to be examined is of poor quality, as finding virus particles can be time-consuming.

Such samples that can be difficult to examine include the supernatants of virus-infected cells [18] as sometimes only a few virus particles are released in the culture medium upon infection. Resin-embedding of the cultured infected cells and ultramicrotomy can address this issue [19], but for more rapid TEM examination, several strategies may be used to concentrate virus-infected cell supernatants [7,20]. First, the sample can be concentrated prior to being deposited on the TEM grid, by purification and/or ultracentrifugation [20]. Secondly, this concentration can be performed on the TEM grid membrane during the sample deposit by (i) using the glow-discharge of the TEM grid to increase electrostatic charges and thus interactions between the virus particles and the membrane [21]; (ii) performing ultracentrifugation directly on the grid membrane with a dedicated system [22]; and (iii) coating the grid membrane with molecules or antibodies to improve adhesion of the viruses to the membrane [23,24]. Lastly, the length of time that the virus sample is deposited can be increased to improve the process. However, although all these methods can be combined [25] in order to concentrate viruses on the TEM grid membrane, it can still be hard to examine virus-poor samples using TEM [20].

In addition to TEM, scanning electron microscopy (SEM) can also be used to visualize virus particles [26,27,28,29]. We have already demonstrated the strengths of SEM for examining samples containing viruses, such as patient swabs [30] and ultra-thin sections of resin-embedded samples containing viruses [31,32]. One advantage of SEM over TEM is the possibility of rapidly screening several large specimens, such as microscopy slides.

Here, we used SEM to analyze microscopy slides and studied different concentration methods for analyzing virus-infected cell supernatants. In order to concentrate the virus-infected cell culture supernatants, we chose SARS-CoV-2 as model virus and tested a panel of coating molecules for microscopy slides as well as the effect of centrifugation. We found that the use of 25X ConcanavalinA lectin coating of 12 mm microscopy glass slides in glass shell vials coupled with ultracentrifugation provided the best method for concentrating SARS-CoV-2-virus-infected cell supernatants for SEM routine examination during viral diagnosis.

## 2. Materials and Methods

### 2.1. Virus Production

The Delta variant of the SARS-CoV-2 IHUMI-3630 strain was used in this study as a model virus. After thawing from −80 °C, the virus sample was diluted to 1/10th in M4 medium consisting of MEM medium supplemented with 4% fetal calf serum and 1% L-glutamine. Vero-E6 cells in 12-well plate were inoculated with 500 µL of this virus solution and incubated for 1 h at 37 °C with 5% CO_2_. The medium was completed to 2 mL and supernatants were collected after further incubation of the inoculated cells for 48 h at 37 °C with 5% CO_2_ corresponding to the appearance of a cytopathic effect. Before being deposited on glass slides, cell culture supernatants were filtrated at 0.2 µm. Polymerase Chain Reaction (PCR) was performed in parallel on cell culture supernatants. The mean Ct value was 13.7 ± 1.3 (n = 7).

### 2.2. Capture and Concentration of Viral Particles

Microscopy Lab-Tek 8-well chamber slides (Lab-TekII 8 well CC2 Glass, 154941PK, ThermoFisher Scientific, France) and 12 mm glass shell vials (080172, Dutscher, Bernolsheim, France) were coated with a panel of both positively charged and negatively charged molecules by depositing 100 µL (Lab-Tek) and 200 µL (shell vials) of these solutions on top of the slides for 60 min at room temperature (RT) in a moist chamber prior to depositing the supernatants. The concentrations of the different coating molecules used in this study were as follows: polyethylene glycol (PEG-it Virus Precipitation Solution 5X, LV825A-1, System Biosciences, Palo Alto, CA, USA) at 1X; poly- D-Lysine (A38904-01, ThermoFisher Scientific, France) at 0.01 mg/mL; poly-L-Lysine (P4832, Sigma-Aldrich, Saint-Quentin-Fallavier, France) at 0.01 mg/mL; ApoH (ApoH protein, PT08011-1MG, ApoH-Technologies, France) at 0.1 mg/mL; Polybrene (Polybrene Transfection Reagent, TR-1003-G, Merck Sigma-Aldrich, Saint-Quentin-Fallavier, France) at 0.1 µg/mL; Sulfatides (Sulfatides Brain, 131305P-10MG, Merck Sigma-Aldrich, France) at 0.1 mg/mL; ConcanavalinA (ConA, 15566286, Fisher Scientific, France) at concentrations of 0.1X, 1X, 5X, 10X, 25X, 75X and 100X, corresponding to concentrations of 250 ng/mL, 2.5 µg/mL, 12.5 µg/mL, 25 µg/mL, 62.5 µg/mL, 187.5 µg/mL and 250 µg/mL, respectively. After being deposited, these coating solutions were discarded and replaced by 100µL (Lab-Tek) and 200 µL (shell vials) of cell culture supernatants for 10 min at RT. Centrifugation of the shell vials was performed for 30 min at 3452 g using a Heraeus Multifuge 3 S-R ultracentrifuge (ThermoFisher Scientific, France).

### 2.3. Scanning Electron Microscopy

For scanning electron microscopy (SEM), deposited glass slides (Lab-Tek and Shell-vial 12 mm) were fixed with glutaraldehyde 2.5% in 0.1 M of sodium cacodylate buffer for 60 min. Slides were rinsed with 0.1 M sodium cacodylate buffer and distilled water for 1 minute each. Slides were dehydrated with increasing ethanol solutions (30%, 50%, 70%, 90%) for 2 minutes and with 100% ethanol for 5 minutes. Slides were incubated with 100% ethanol/100% hexamethyldisilazane (HDMS) in a 1:2 ratio for 5 minutes. Slides were incubated with 100% of HDMS for 5 minutes and air-dried for 30 min. We checked that no particles had been lost after this virus preparation (dehydration and drying) for SEM (not shown). Finally, the slides were platinum sputter-coated for 20 s at 10 mA (Hitachi MC1000). The observation was made using a SU5000 (Hitachi High-Technologies, Tokyo, Japan) SEM with an SE detector in high-vacuum mode at 1 kV acceleration voltage, observation mode (spot size 30). The working distance ranged between 1 mm and 5 mm. For quantification, automatic 6 × 6 mosaic tiled images at ×5k magnification were acquired at a random position with auto contrast/brightness using the microscope zig-zag function after manual adjustment of the focus and no further auto-focus. Pixel size was 3.96 nm. Images were 1280 × 960 pixels corresponding to 5078 × 3808 nm fields of view.

### 2.4. Immunocytochemistry and Fluorescence Confocal Laser Scanning Microscopy

For immunocytochemistry, supernatant-deposited slides were fixed with 4% paraformaldehyde for at least 20 min at RT alongside slides fixed for SEM from the same experiments. After a single rinse in Phosphate Buffer Saline (PBS), slides were saturated with 10% Fetal Bovine Serum (FBS) and 0.1% Tween 20 in PBS for 10 min at 37 °C. Slides were incubated for 1 hour at 37 °C with a polyclonal rabbit anti-SARS Coronavirus Spike protein (1:1000, PA5-81795, ThermoFisher, France.) The slides were washed twice with PBS and incubated for 30 min at 37 °C with an Alexa Fluor 488-Goat anti-Rabbit IgG (H + L) secondary antibody (A11008, ThermoFisher Scientific, France). The slides were rinsed twice in PBS then mounted with Moviol for observation. Images were acquired using a LSM800 confocal laser scanning microscope (Zeiss) with a Plan-Apochromat 63X/1.4 oil objective. Scan zoom was 2.4. Pixel dwell time was 4.12 µs. Averaging was 2. Image dimensions were 512 × 512 pixels, 42.26 × 42.26 µm. Pixel size was 83 nm. Mosaics of 6 × 6 tiled images were randomly acquired on each slide, representing 2816 × 2816 pixels and 232.4 × 232.4 µm fields of view.

### 2.5. SARS-CoV-2-like Particle Quantification and Image Processing of SEM and CLSM Images

Quantification of the abundance of SARS-CoV-2-like virus particles in the SEM x25k images was performed manually upon visual inspection. The number of deposited SARS-CoV-2-like particles was counted and was considered as the 100% deposition amount for comparisons. Counting was also carried out in parallel with a macro created with Fiji [33] in order to depict SARS-CoV-2-like particles, by batch processing images from a folder with: run(“Set Scale...”, “distance = 504.3 known = 2000 unit = nm global”); run(“Auto Threshold”, “method = Yen white”); run(“Median...”, “radius = 6”); run(“Invert”); setOption(“BlackBackground”, false); run(“Make Binary”); run(“Analyze Particles...”, “size = 6000–20000 circularity = 0.85–1.00 show = Outlines display exclude summarize record add in_situ”). The average percentage error of this automated counting was estimated to 14% and thus was used only for preliminary counts. We also developed a macro in Fiji dedicated to quantification of all virus-like round particles with diameters ranging from 5 nm to 200 nm, such as batch processing: run(“Set Scale...”, “distance = 504.3 known = 2000 unit = nm global”); //setTool(“rectangle”); makeRectangle(0, 0, 1280, 900); run(“Crop”); run(“Auto Threshold”, “method = Yen white”); run(“Median...”, “radius = 6”); run(“Invert”); setOption(“BlackBackground”, false); run(“Make Binary”); run(“Analyze Particles...”, “size = 100–1000000 circularity = 0.85–1.00 show = Masks display exclude summarize record add in_situ”). Rectangle cropping is optional in the latter macro and used when annotations are merged into the image, such as scale and microscope settings. Based on the objects listed after processing this macro on x25k images from tile acquisitions, a Feret distribution of object diameters was performed. To quantify virus abundancy in confocal microscopy images, 6 × 6 tile CLSM images were exported as black and white 16-bit tiff images. After scaling and thresholding at 6000 intensity value, the image contrast was inverted and signal fluorescence quantified by white pixels counting with histogram analysis over the whole image.

## 3. Results

### 3.1. Scanning Electron Microscopy (SEM) of SARS-CoV-2-like Particles from SARS-CoV-2-Infected Cell Culture Supernatants

**Figure 1:**
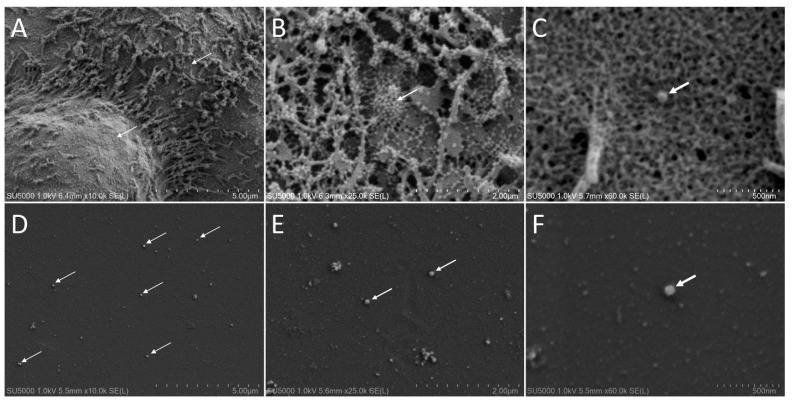
Scanning electron microscopy of SARS-CoV-2-like particles. (**A**–**C**): Secondary electron detector and 1 kV acceleration voltage used for imaging whole SARS-CoV-2 VeroE6-infected cells after fixation, dehydration and HDMS drying. (**D**–**F**): Secondary electron detector and 1 kV acceleration voltage used for imaging SARS-CoV-2 VeroE6 infected cell supernatants, after fixation, dehydration and HDMS drying. Arrows point to SARS-CoV-2 particles.

The use of SEM for imaging whole virus particles is comparable to the TEM negative staining technique, providing morphological features and measurable dimensions (Golding et al. 2016). As a result of preliminary experiments on SARS-CoV-2-infected cell monolayers cultured in Lab-Tek chambers (Figure 1A–C), we chose the secondary electron (SE) detector and a very-low acceleration voltage (1 kV) as the best imaging set-up for observing SARS-CoV-2-like particles from culture supernatants by SEM (Figure 1D–F). Figure 1 illustrates SARS-CoV-2-like particles located on SARS-CoV-2-infected cells (Figure 1A–C) and from SARS-CoV-2-infected cell supernatants (Figure 1D–F). In SARS-CoV-2-infected cell monolayers, SARS-CoV-2 virion particles could be depicted, decorating apical domains of cells. SARS-CoV-2-like virus particles appear as round to slightly squared, well contrasted, 90–100 nm in diameter objects. These criteria were in accordance with the literature and were further used for analyzing cell culture supernatants [13,14,26,28,29,34]. A magnification of ×5k was chosen as it provided the best compromise between large fields of view and morphological depiction of the deposited objects, especially SARS-CoV-2-virus-like particles.

### 3.2. Testing of Different Coating Molecules for the Capture of SARS-CoV-2 Virus Particles on Lab-Tek

As potential adhesive molecules for capturing SARS-CoV-2 viruses, we chose both theoretically negatively and positively charged coating molecules. The negatively charged molecules were (1) polyethylene glycol (PEG) [35]; (2) sulfatides [36]; ConcanavalinA (ConA) [37,38]. Positively charged molecules were 1) polybrene [39]; 2) ApoH [40]; (3) poly L- and D- Lysine [41]. As a result of the preliminary experiments, we checked that the coating of the glass slides with these molecules did not induce artifacts seen by SEM that could eventually interfere with the depiction of the virus particles (not shown).

**Figure 2:**
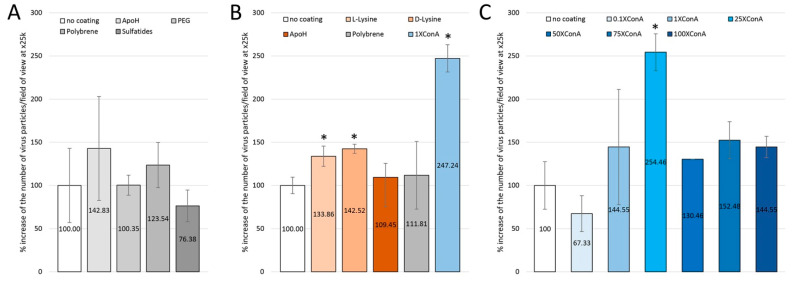
SEM quantification of the abundancy of SARS-CoV-2 virus-like particles after deposition of VeroE6 infected cell supernatants on Lab-Tek glass slides. (**A**): Comparison of abundancy of SARS-CoV-2-like particles between no coating and ApoH, PEG, Polybrene or Sulfatides coating conditions. (**B**): Comparison of abundancy of SARS-CoV-2-like particles between no coating and poly-L-lysine, poly-D lysine, ApoH, Polybrene and 1XConA coating conditions. (**C**): Comparison of abundancy of SARS-CoV-2-like particles between no coating and 0.1XConA, 1XConA, 25XConA, 50XConA, 75XConA and 100XConA coating conditions. Error bars represent standard deviations. *: *p*-value < 0.05, Student’s *t*-test.

In a first round of three independent experiments (one slide for each condition in three experiments; 36 fields of view per slide), we quantified the amount of SARS-CoV-2-virus-like particles deposited on non-coated slides compared to ApoH-, PEG-, polybrene- and sulfatide-coated slides (Figure 2A). In these experiments, the number of deposited particles was 1.38 ± 0.59 per x25k field of view on the non-coated slides, 1.97 ± 1.19 on the ApoH-coated slides, 1.38 ± 0.16 on the PEG-coated slides, 1.70 ± 0.45 on the polybrene-coated slides and 1.05 ± 0.19 on the sulfatide-coated slides. Thus, there was no significant increase in these independent triplicates in terms of the amount of deposited particles, as was also the case for triplicates within another experiment (three slides for each condition; not shown). In a second experiment with triplicates (three slides for each condition; 36 fields of view per slide), we quantified the amount of deposited SARS-CoV-2-virus-like particles on non-coated slides compared to slides coated with Poly-L-Lysine and Poly-D-Lysine, ApoH, polybrene and ConA (Figure 2B). In this experiment, the number of deposited particles was 1.18 ± 0.11 on the non-coated slides, 1.57 ± 0.18 on the poly-l-lysine-coated slides, 1.68 ± 0.09 on the Poly-d-Lysine-coated slides, 1.29 ± 0.21 on the ApoH-coated slides, 1.31 ± 0.52 on the polybrene-coated slides and 2.91 ± 0.46 on the 1XConA-coated slides. There was a significant increase in the amount of deposited particles for Poly-L-Lysine and Poly-D-Lysine coatings, with 34% and 43% increases, respectively, and for the 1XConA coating, for which the increase was 147% (n = 3; Student’s *t*-test). Next, we asked whether we could further improve the efficiency of the deposit with different concentrations of the ConA solution. We compared 0.1X, 1X, 25X, 50X, 75X and 100X concentrated ConA coating solutions to no-coated slides and we found a peak increase of 154% in particle deposition with ConA at 25X concentration (Figure 2C) (n = 3, except for 50XConA one slide only; Student’s t-test). Indeed, the number of deposited particles was 0.94 ± 0.26 on the non-coated slides, 0.63 ± 0.13 on the 0.1XConA-coated slides, 1.35 ± 0.90 on the 1XConA-coated slides, 2.38 ± 0.51 on the 25XConA-coated slides, 1.22 on the 50XConA-coated slides, 1.43 ± 0.31 on the 75XConA-coated slides and 1.35 ± 0.17 on the 100XConA-coated slides.

We also tested whether doubling the volume of the deposited cell culture supernatant or doubling the deposition time would increase the deposit of virus particles. In neither case was the effect significant, with 36% and 11% increases, respectively.

### 3.3. Testing of Centrifugation for SARS-CoV-2 Virus Particle Capture in Shell Vials

Centrifugation can prove useful for concentrating virus particles on TEM grids as a result of dedicated devices (Hammond et al. 1981). Because the device was found to be too complex to handle for routine SEM observation of the use of TEM grids, we chose to perform centrifugation of shell vials, glass tubes classically used in our NSB3 laboratory for culturing virus-infected cells, containing 12 mm microscopy glass slides.

**Figure 3:**
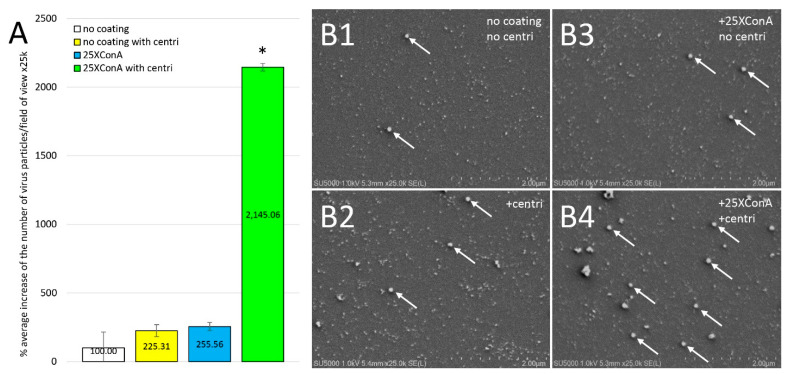
Effect of centrifugation and 25XConA coating on the deposition of SARS-CoV-2-like particles in shell vials. (**A**): Quantification of the amount of SARS-CoV-2-like particles deposited on non-coated 12 mm glass slides with and without centrifugation and of the amount of SARS-CoV-2-like particles deposited on 25XConA-coated 12 mm glass slides, with and without centrifugation. (**B**): SEM images of single fields of view at x25k after depositing SARS-CoV-2-infected cell culture supernatant, with (**B2**,**B4**) and without (**B1**,**B3**) centrifugation and with (**B3**,**B4**) and without (**B1**,**B2**) 25XConA coating. Error bars represent standard deviations. *: *p*-value < 0.05, Student’s *t*-test. The arrows point to Sars-CoV-2 virus-like particles.

We observed a significant 125% increase in the amount of deposited SARS-CoV-2-like particles on non-coated 12 mm glass slides after performing centrifugation, with 0.68 ± 0.30 versus 0.30 ± 0.35 particles per field of view, respectively (Figure 3A,B1,B2) (n = 3; Student’s *t*-test). When adding centrifugation to the 25XConA-coating, we obtained a 1145% increase in the deposit of SARS-CoV-2-virus-like particles, with 6.44 ± 1.80 versus 0.77 ± 0.23 particles per field of view, respectively (Figure 3A,B3,B4) (n = 3; Student’s *t*-test).

### 3.4. CLSM Microscopy of Fluorescently Stained SARS-CoV-2-Infected Cell Culture Supernatants after Concentration in Shell Vials

**Figure 4:**
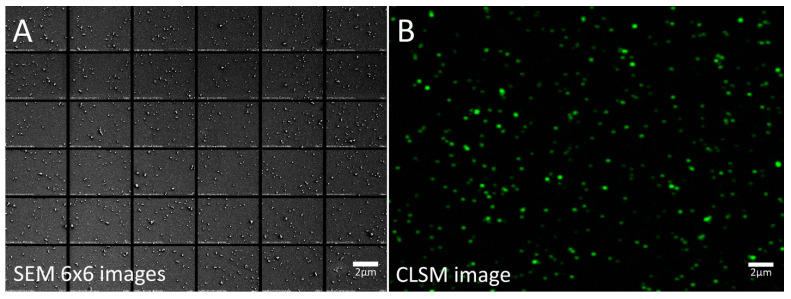
SEM and CLSM images viewed at the same scale of two different regions SARS-CoV-2-infected cell culture supernatants after 25XConA coating and centrifugation. (**A**): SEM 6 × 6 mosaic 1280 × 960 pixel images at x25k magnification. (**B**): Zoom-in on a single 512 × 512 pixel CLSM image of Alexa-488 fluorescence signal after anti-SARS-CoV-2 staining at ×63 magnification, zoom 2.4.

**Figure 5:**
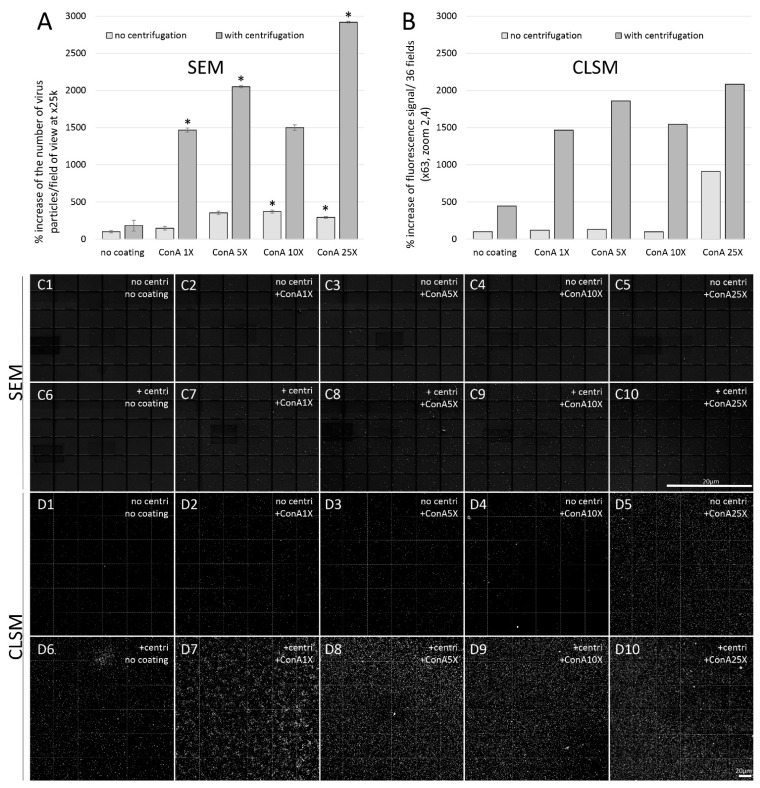
Quantification of the amount of SARS-CoV-2-like particles by SEM (**A**) and of fluorescence signal after anti-SARS-CoV-2 immunostaining by CLSM (B) and respective corresponding images (**C**,**D**). Deposition of SARS-CoV-2-infected cell culture supernatants after +/− centrifugation on non-coated slides and slides coated with 1X, 5X, 10X and 25X ConA. (**C1**–**C10**): SEM 6 × 6 mosaic images at x25k magnification; (**D1**–**D10**): binary CLSM 6 × 6 mosaic images at x63/zoom 2.4 magnification after threshold. Error bars represent standard deviations. *: *p*-value < 0.05, Student’s *t*-test. Scale bars in (**C10**,**D10**) for SEM and CLSM images, respectively.

In order to confirm that SARS-CoV-2-like particles observed by SEM were indeed SARS-CoV-2 virus particles, we prepared in parallel in the same experiment +/− ultracentrifuged +/− ConA-coated deposited shell-vial glass slides (i) for SEM and (ii) for fluorescence microscopy after anti-SARS-CoV-2 fluorescent staining (Figure 4 and Figure 5). Figure 4 illustrates the difference between the two imaging modalities, EM versus LM: for the same field of view/surface area, Sars-CoV-2-virus-like particles seen indirectly after immunostaining (Figure 4B) appear larger in size and fewer in number than by direct SEM observation (Figure 4A). This difference may originate from the lower capacity of CLSM compared to SEM to discriminate the smallest objects (lower resolution) as well as from the fact that not all particles visible by SEM are actually Sars-Cov-2 virus particles. The SEM examination was performed as previously illustrated (Figure 5C). Confocal laser scanning microscopy (CLSM) was used to acquire 6 × 6 tile scans of immunostained slides after using a primary anti-SARS Coronavirus Spike protein and a secondary fluorescent antibody (Figure 5D). Negative controls were performed in order to check the specificity of our immunostainings, by using the supernatant of non-infected cells and by omitting primary anti-SARS-CoV-2 antibodies in our staining protocol of SARS-CoV-2-infected cell supernatants. Neither case showed significant staining (not shown). The fluorescence signal of background pixels was indeed negligible in negative controls, with an intensity around 6000 (16-bit images), corresponding to the threshold value that we used for signal quantification from our datasets.

Our quantifications for each condition of the amount of particles counted by SEM and of the total fluorescence signal by CLSM revealed very similar increases, both regarding the trend of increase when increasing ConA concentration and the amount of increase for each condition (Figure 5). Indeed, with a total of 7,929,856 pixels for each CLSM 6 × 6 tile scan, we found the following numbers of white pixels (fluorescence signal): without centrifugation, 41,462 with no ConA-coating, 49,220 (+19% increase) with 1XConA-coating, 54,136 (+31%) with 5XConA-coating, 40,558 (-2%%) with 10XConA-coating and 377,779 (+811%) with 25XConA-coating; with centrifugation, 184,581 (+345% increase) with no ConA-coating, 607,899 (+1366%) with 1XConA-coating, 771,039 (+1760%) with 5XConA-coating, 640,206 (+1444%) with 10XConA-coating and 864,251 (+1984%) with 25XConA-coating (Figure 5A). In parallel, using SEM we found the following mean numbers of particles per field of view: without centrifugation, 12.33 ± 2.08 with no ConA-coating, 18.00 ± 4.58 (+46% increase) with 1XConA-coating, 43.50 ± 10.61 (+253%) with 5XConA-coating, 45.67 ± 8.96 (+270%, significant) with 10XConA-coating and 36 ± 4.58 (+192%, significant) with 25XConA-coating; with centrifugation, 22.33 ± 16.17 (+81% increase) with no ConA-coating, 181 ± 51.03 (+1368%, significant) with 1XConA-coating, 253 ± 38.43 (+1951%, significant) with 5XConA-coating, 185 ± 70.71 (+1400%) with 10XConA-coating and 360 ± 42.04 (+2819%, significant) with 25XConA-coating (n = 3; Student’s *t*-test) (Figure 5B).

Thus, CLSM datasets confirmed that SARS-CoV-2 virus-like particles in our SEM images that we counted in our experiments were indeed likely to be SARS-CoV-2 virus particles.

### 3.5. Perspectives for Automated Processing of SEM and CLSM Images of SARS-CoV-2-Infected Cell Culture Supernatants

Our SEM images of SARS-CoV-2 virus particles from infected cell culture supernatants provide very suitable images for automatic particle analysis due to the high contrast. One potential issue would be the aggregation of particles, which may pose difficulties for depicting single virus particles. In our experiments, we did not notice a substantial aggregation of Delta SARS-CoV-2-virus-like particles originating from cell supernatants, which was shown for retroviral particles pseudotyped with the Delta variant SARS-CoV-2 Spike protein [42].

**Figure 6:**
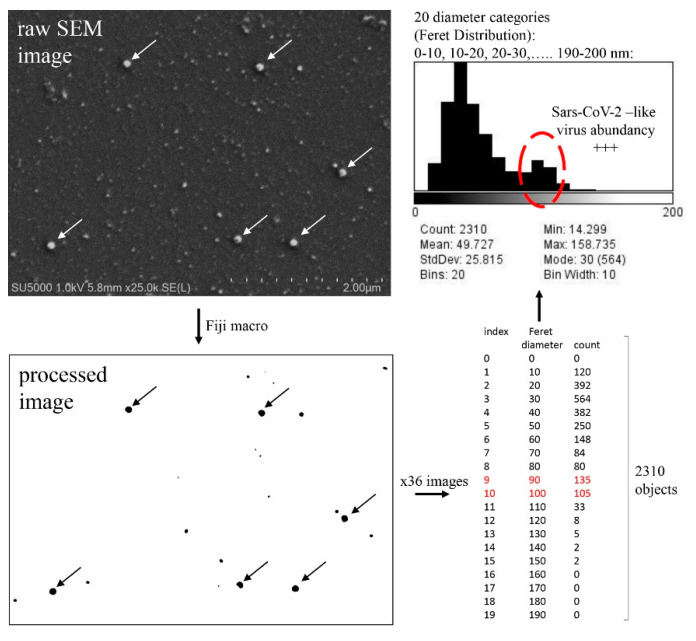
Example of Fiji macro for depicting 5 nm to 200 nm diameter particles in SEM images of SARS-CoV-2-infected cell culture supernatants. In this example SARS-CoV-2-infected cell culture supernatant with Ct value of 13.72 was concentrated by centrifugation on a 25XConA slide in a shell vial. Arrows point to Sars-CoV-2 virus-like particles in raw SEM and processed images. +++: peak.

We used a targeted Fiji macro dedicated to highlighting and counting SARS-CoV-2 particles for preliminary quantifications of our SEM images folders (see Section 2.5). We also developed a macro (Figure 6; see Section 2.5) aimed at depicting all round virus-like particles with diameters ranging from 5 nm to 200 nm.

Such macros may be adapted and used for estimating non-viral background materials from cell culture supernatants after 0.2 µm filtration as well as for discovering the presence of a known or unknown virus in a suspected sample. Finally, in the present study, we chose 512 × 512 pixels at x63/zoom 2.4 magnification (80 nm pixel size) for quantifying fluorescence signal of anti-SARS-CoV-2 stained supernatants by CLSM. For counting single SARS-CoV-2 virus particles, 4096 × 4096 pixel images at ×63/zoom 2.4 magnification (10 nm pixel size) would be more accurate but more time-consuming to acquire.

## 4. Discussion

**Figure 7:**
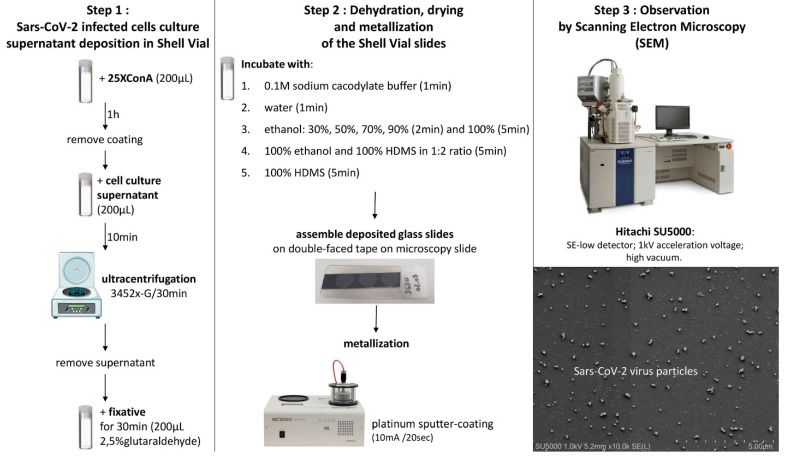
Recapitulative scheme for improved viral diagnosis of SARS-CoV-2-infected cell culture supernatants by scanning electron microscopy.

In the following scheme (Figure 7), we summarize the methodological steps for concentrating SARS-CoV-2-infected cell culture supernatants and detecting virus-like particles by scanning electron microscopy.

The total duration of our method with ready to use coated glass-slides represents around 2 h and 20 min in total for assessing virus-like particle presence: 10 min for cell culture supernatant deposition, 30 min for ultracentrifugation, 30 min for fixation, 1 h for washes, dehydration, drying, slides mounting and metallization and 10 min for SEM examination.

Our method was primarily designed for bringing a yes/no answer to the question of the presence of virus-like infectious particles, especially for poor cell culture supernatants. It also opens perspectives for quantitative considerations about the viral charge of a cell culture supernatant, which may be of interest when performing pharmacological studies or comparing variants. Compared to real-time polymerase chain reaction (PCR) experiments, our method does not bring advantages for monitoring viral charge in case of known viruses. However, in the case of unsuspected or unknown new viruses, our method can provide a monitoring of the amount of virus-like particles located in an inoculated cell culture, making our method of interest for emergent viral infections, even with low-replicating viruses, thus contributing to DEM [2].

Regarding the question of the qualitative morphological characterization of virus-like particles, i.e., virus identification, our method is limited by several points: (i) the metallization process that adds thickness to nano-objects, thus deforming them and limiting observation of fine details, such as Sars-Cov-2 spikes; (ii) our method would also benefit from critical-point-drying that would better preserve particles morphology; and (iii) higher magnifications would improve morphological depiction. Nevertheless, our imaging setup in our method can be used to discard potential viruses according to shape and diameter and refine virus class. Our method may thus be used as a routine first-line strategy for EM viral diagnosis of suspects samples, with more in-depth second-line morphological analysis using TEM, for example. Potential improvements and greater efficiency when performing targeted LM immunostainings may arise from super-resolution light microscopy [43] and the use of gridded 12 mm glass slides in shell vials for correlative light electron microscopy (CLEM) at selected regions of interest [44].

Machine learning would also be of high interest for automating detection and quantification of virus-like particles in SEM ±CLSM images [45,46] as well as for virus identification and DEM. The creation of reference databases with known viruses are needed for this later point, and our method illustrates one strategy that may be used to create such databases, with automatic acquisitions and large datasets. Finally, centrifugation may be combined with other coating molecules, such as lectins other than concanavalin-A for better Sars-CoV-2 capture [47,48] or with other coating reagents for the targeted concentration of other model viruses.
